# Case of Luxation of the Knee-Joint

**Published:** 1858-01

**Authors:** C. Hixon

**Affiliations:** Petersburg, Indiana


### ART. VII.—*A Case of Luxation of the Knee-Joint.* By C. HIXON, M.D., of Petersburg, Indiana.

DISLOCATION of the knee-joint, from its rare occurrence and serious nature, is deprecated by the surgeon into whose hands so formidable an injury may chance to fall. On account of the extensive surfaces of the bones entering into and forming the articulation, together with the firm manner in which they are secured by ligaments, both within and without the joint, a considerable amount of force must necessarily be brought to act upon the parts before a dislocation can be effected.

Judging from the anatomy of the parts entering into the formation of the knee-joint, a dislocation must necessarily be productive of serious injury to the soft parts, and such, I presume, will generally be found to be the case. Nor is the extent of the injury in itself, and the obstacles which may oppose a speedy reduction, all the sources of disquiet to the patient or surgeon. The prospect of an amputation, on account of gangrene following laceration of the popliteal vessels, or from protracted suppuration of the joint from destructive inflammation on the one hand, and the consequences of such serious mutilation on the other, are indeed adequate causes of disquiet to both.

The following case, which occurred in my practice recently, from the extensive injury to the parts, together with the favorable termination of the case, may, perhaps, be a sufficient apology for presenting it to the profession :—

A middle-sized, muscular man was forcibly thrown from a sleigh in February, 1856, causing a very serious dislocation of his right knee-joint; the head of the tibia was thrown forward and upward, the inferior extremity of the femur being lodged low on the posterior side of the tibia, the ends of the two bones having passed about five inches. All the ligaments, except the ligamentum patellæ, were believed to be ruptured. Extensive injury was also done to the popliteal vessels, as there was considerable extravasation of blood about the joint, and persistent coldness of the foot and leg.

Little resistance was offered to reduction. To an assistant was intrusted the charge of fixing firmly the thigh, while, by means of a lac around the ankle, extension was made, approximating toward an angle of about thirty degrees. While extension was thus slowly but steadily made, I placed my hands, locked, under the end of the femur, and pulled gently in a forward direction, when the ends of bones came easily to their proper position. Perhaps less resistance was offered to reduction in this case than usual, from the fact of my patient's fears in regard to an amputation being so powerfully excited as almost to cause syncope, and which I took occasion, designedly, to increase rather than abate, thus obviating the necessity for the use of the lancet, tobacco, or chloroform. As no appropriate surgical apparatus was at hand, (it being 10 o'clock at night, and about eight miles from town,) a roller was applied with moderate firmness to the limb, the latter bound to its fellow, and a pillow under the joint to keep it flexed sufficiently to prevent its again slipping out. It was thus suffered to remain until next day, when an inclined plane was adjusted to the limb.

The treatment consisted in the application of evaporating lotions, perfect rest of the limb, mild aperients, restricted diet, and opium to allay consecutive irritation. In four weeks the apparatus was removed and moderate working of the joint permitted. A strong leather cap was also laced firmly around the joint, to give support to the parts, and the patient allowed to go upon crutches.

The case thus progressed without any unfavorable symptoms, and the patient is now able to walk without any artificial support whatever, and to perform his daily labor as usual.

